# Research progress on metabolites of nitrofurazone in aquatic products

**DOI:** 10.1016/j.heliyon.2024.e29735

**Published:** 2024-04-16

**Authors:** Guangxin Yang, Shuhai Ding, Junyu Zhang, Lin Gu, Wenlei Zhai, Cong Kong

**Affiliations:** a (East China Sea Fisheries Research Institute, Chinese Academy of Fishery Sciences, Shanghai, China; b (School of Environment and Architecture, University of Shanghai for Science and Technology, Shanghai, China; c (Institute of Quality Standard and Testing Technology, Beijing Academy of Agriculture and Forestry Sciences, Beijing, China

**Keywords:** Nitrofurazone, Semcarbazide, Detection methods, Aquatic crustacean, Potential metabolites

## Abstract

The carcinogenic and teratogenic risks of nitrofurazone (NFZ) led to its restriction in aquatic products. Semicarbazide (SEM), one of its metabolites, is a primary focus of modern monitoring techniques. However, the SEM residue in aquatic products is believed to be formed through endogenous mechanisms, especially for aquatic crustaceans. In this article, we will discuss the source of SEM, including its usage as an antibiotic in aquatic products (nitrofurazone), its production during food processing (azodicarbonamide and hypochlorite treatment), its occurrence naturally in the body, and its intake from the environment. SEM detection techniques were divided into three groups: derivatization, extraction/purification, and analytical methods. Applications based on liquid chromatography and its tandem mass spectrometry, immunoassay, and electrochemical methods were outlined, as were the use of various derivatives and their assisted derivatization, as well as extraction and purification techniques based on liquid-liquid extraction and solid-phase extraction. The difficulties of implementing SEM for nitrofurazone monitoring in aquatic products from crustaceans are also discussed. Possible new markers and methods for detecting them are discussed. Finally, the present research on monitoring illicit nitrofurazone usage through its metabolites is summarised, and potential problems that need to be overcome by continuing research are proposed with an eye toward giving references for future studies.

## 1 Introduction

**Fig. 1:**
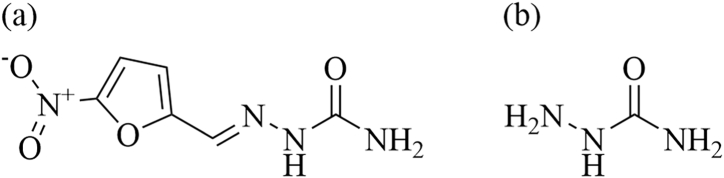
Molecular structure of nitrofurazone (a) and its metabolite, semicarbazide (SEM) (b).

The development of modern fishery has significantly improved the living standards of the public. With the increasing demand for aquatic products, their quality and safety issues have also attracted much attention. Nitrofurazone (NFZ) (Fig. 1a) is a broad-spectrum antibiotic that belongs to nitrofuran drugs. It has antibacterial effects on a variety of Gram-positive and negative bacteria and has been used to treat bacterial diseases in aquaculture. However, the International Agency for Research on Cancer (IACR) classified NFZ and its metabolite semicarbazide (SEM) (Fig. 1b) as class III carcinogens in 1987 and 1990 [1]. NFZ has potential teratogenicity and carcinogenicity. It can be exposed to humans through the food chain. The European Union, the United States, and China have consecutively prohibited the use of several veterinary medications. In 2002, NFZ was included in the list of 11 drugs banned in imported animal-derived foods published by the US Food and Drug Administration. The Ministry of Agriculture in China also announced the prohibition of the use of NFZ in 2002 [2]. In 2003, the European Union passed the detection method of nitrofuran metabolites in aquatic products, and the detection limit should not be higher than 1 μg/kg [3].

NFZ is a light-sensitive drug with a short half-life. Its parent compound is rapidly metabolized in animal bodies and drops below the limit of detection quickly. However, its metabolite, SEM, is a confirmed marker of drug use and can remain in the body for months in a protein-bound state. SEM has diverse sources, including endogenous SEM in aquatic crustaceans and its intentional use during aquaculture. Furthermore, the processing of aquatic products and environmental accumulation can be potential sources of SEM. Non-drug sources pose a risk of misjudgment in monitoring the illegal use of NFZ in aquatic products. Recent reviews on monitoring NFZ and its metabolite residues in aquatic products have focused on the sources and detection techniques of the metabolites, with less attention given to pretreatment techniques and potential alternative metabolites [4,5]. Thus, this article provides a comprehensive review of research progress on the sources of SEM, pretreatment techniques, detection methods, relevant detection risks, and potential other residue markers of NFZ both domestically and internationally over the past five years. This review aims to provide a further reference for the accurate monitoring of SEM residue and the use of NFZ in aquatic crustaceans.

## 2 SEM in aquatic products

**Fig. 2:**
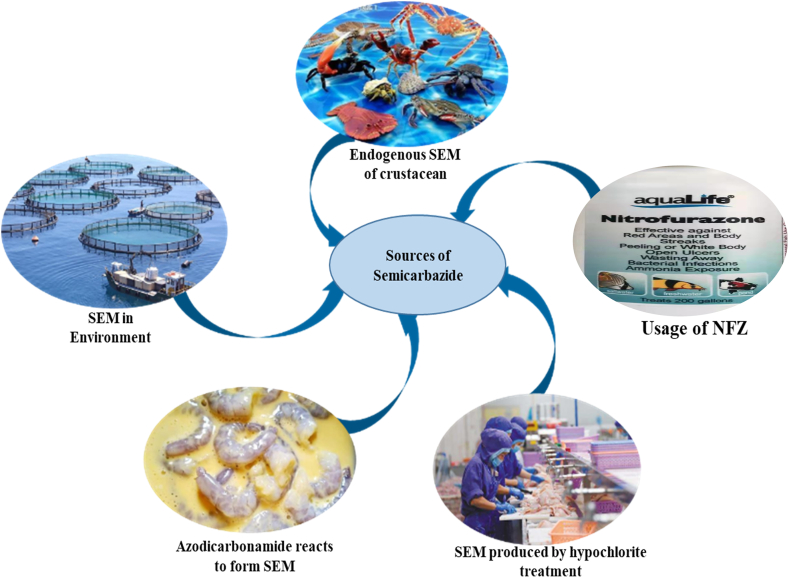
Schematic diagram of the possible sources of SEM in aquatic products.

In recent years, research has indicated that there are five possible pathways for the presence of SEM residues in aquatic products (as depicted in Fig. 2): illegal use of NFZ drugs, endogenous production in aquatic crustacean, environmental accumulation, processing during chlorination disinfection, and co-processing with raw materials containing dimethylamine. Each of these pathways will be discussed in further detail below.

### 2.1 Usage of NFZ

Although NFZ has been banned in aquaculture, it is still used repeatedly because of its excellent antibacterial effect. SEM is a metabolite of NFZ and is considered to be a marker for judging whether NFZ is abused in animal-derived foods. Due to the severe harm of such drugs, the EU has banned nitrofuran drugs in edible livestock, poultry, and aquatic animals since 1995. Then the United States, South Korea, and other countries have also adopted relevant detection and control measures. Due to the establishment of relevant EU detection and control measures, China's aquatic product trade industry has caused significant losses, and China has also issued relevant bans.

With the improvement of relevant laws and regulations, the use of nitrofuran drugs in the domestic aquaculture industry of China has been significantly reduced, and the detection of SEM in exported aquatic products is rare. However, due to the lack of implementation and supervision of laws and regulations and the lack of integrity of farmers, there are still negative reports of drug residues detected by farmers due to the use of nitrofuran drugs in China.

### 2.2 Endogenous SEM of crustacean

SEM residues can be present not only due to the illegal use of NFZ, but also through endogenous pathways in some aquatic crustaceans, where it accumulates in tissues [6]. Yu et al. [7] monitored SEM levels in different stages of *vannamei* shrimp growth and found that SEM was present in shrimp shells but not in muscle tissue, indicating that SEM originates from the shrimp shells. Zhang et al. [8] studied SEM residues in crustacean aquatic products from Zhejiang, China, and found that the SEM content in the muscle, shell, and internal organs of different species of shrimp and crab ranged from 0.35 to 26.62 μg/kg and exhibited tissue specificity. Yu et al. [9] compared the levels of SEM residues in fried, steamed, and baked shrimp with those in unprocessed shrimp and found that processed shrimp had higher levels of SEM residues. Factors such as temperature, moisture, and oil content can affect the formation of SEM, which is likely to be produced by amino acid components at high temperatures. McCracken et al. [10] suggested that shrimp may accumulate SEM through the process of converting and enriching amino acids from algae or other organisms. Existing literature indicates that SEM occurs naturally in various aquatic crustaceans and varies in concentration between tissues. The baseline residue levels of SEM in some aquatic crustaceans are shown in Table 1.

**Table 1:** Background residue of SEM in crustacean aquatic products.

Samples	The content of SEM(μg/kg)		References
	muscle	Carapace	
*Macrobrachium nipponense*	17.9 ± 0.38	26.62 ± 4.46	[8]
*Macrobrachium rosenbergii*	2.12 ± 0.07	24.73 ± 3.10	[8]
*Procambarus clarkia*	2.20 ± 0.35	13.84 ± 2.7	[8]
*Scylla serrata*	N/A	12.6	[11]
*Nephrops norvegicus*	<0.5	<0.5	[11]
*Somanniathelpusa sinensis*	0.6	8.4	[11]
*Penaeus Vanmamei*	N/A	3.94	[7]

### 2.3 SEM produced by hypochlorite treatment

Sodium hypochlorite is a popular disinfectant in the food processing industry due to its low cost, strong activity, and excellent sterilization capabilities. However, using sodium hypochlorite on high-protein products can result in residual of SEM. If treated with sodium hypochlorite, SEM can be produced via the Hofmann reaction at high pH. Possible pathways for introducing SEM into aquatic products through sodium hypochlorite include 1. disinfecting aquatic products with sodium hypochlorite; 2. washing aquatic products with water treated with sodium hypochlorite; 3. residual sodium hypochlorite on production equipment. Zhang et al. [8] investigated the effect of sodium hypochlorite disinfection on the SEM concentration in *Litopenaeus vannamei, Paralithodes camtschaticus,* and *Portunus trituberculatus*, and found that the SEM content in *Litopenaeus vannamei* and *Paralithodes camtschaticus* reached maximum values (196.40 μg/kg and 39.90 μg/kg, respectively) at an effective chlorine concentration of 6 %, while the SEM content in Portunus trituberculatus reached 110.80 μg/kg at 12 % effective chlorine concentration. Abernethy [12] speculated that carbamate ions in hypochlorite solution may react with ammonia or acidic amides in aquatic products to form hydrazine. The hydrazine reacts with urea and other compounds through the urea cycle to form SEM.

### 2.4 SEM in the environment

According to current research, human activities are believed to be responsible for the production of SEM in both aquaculture water and the natural environment, leading to its accumulation in organisms [13]. Xing et al. [14] investigated the accumulation, chemical forms, and distribution of SEM in scallop tissues. They found that SEM accumulates in both free and bound states and can be absorbed from seawater. Islam et al. [15] found high levels of SEM residue in sediment and water samples from many shrimp farms. It might be originated from contaminated feed used in aquaculture, poultry feces fed with contaminated feed during mixed feeding, and the introduction of polluted water bodies around the farm. Tian et al. [16] monitored the spatial and temporal distribution and pollution levels of SEM in seawater, sediment, and organisms in the western Laizhou Bay of Shandong Province. They found that the residual amount of SEM in crustacean aquatic products increased with the concentration of SEM in seawater, with the limit of detection ranging from 0.32 to 0.46 μg/kg.

### 2.5 Reaction with azodicarbonamide to form SEM

**Fig. 3:**
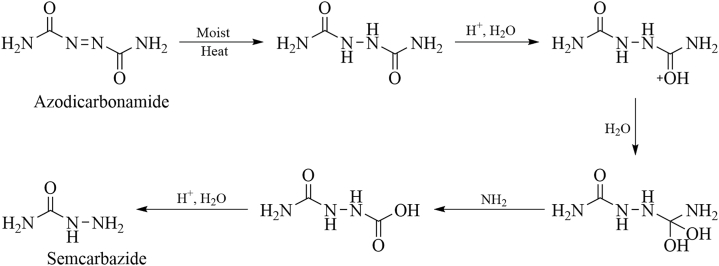
The conversion of SEM from azodicarbonamide.

Azodicarbonamide (ADC) is a commonly used additive in flour that itself does not react with flour and has low acute toxicity. However, when it acts as an oxidant with wet flour, it can produce SEM (as shown in Fig. 3). Pereira et al. [17] detected about 2∼5 μg/kg SEM in flour containing ADC additives. Therefore, when cooking dishes, some aquatic products such as shrimp and crab are coated with flour, which will cause SEM contamination. Yao et al. [18] found that the ADC added to the flour after high-temperature baking would be decomposed into SEM, and the SEM concentration outside was higher than that inside the flour product, indicating that the temperature positively affected the formation of SEM. Therefore, during the processing and transportation, aquatic products should avoid contact with ADC to prevent SEM contamination.

## 3 The detection of SEM

Currently, the usage of NFZ in aquatic products is primarily monitored by detecting the presence of its metabolite, SEM, in residual amounts. Most of the SEM exists in a protein-bound form within biological tissues. Typically, it is hydrolyzed under acidic conditions to release SEM from the sample and then derivatized for extraction, purification, and determination. Commonly used detection methods include high-performance liquid chromatography [19,20], liquid chromatography-tandem mass spectrometry [21,22], immunoassay methods [23], and electrochemical detection methods [24] (as shown in Table 2).

**Table 2:** Methods for the detection of SEM.

Detection method	Advantages	Drawbacks	References
High-Performance Liquid Chromatography(HPLC)	High sensitivity, precision, and accuracy.	Cumbersome operation, complex sample processing, high cost and professional analysts are needed.	[25]
High-Performance Liquid Chromatography-Tandem Mass Spectrometry (HPLC-MS/MS)	High precision, accurate qualitative and quantitative, strong anti-interference ability, high sensitivity, etc.	Expensive instruments, complex operation, long cycle, and professional analysts are needed.	[26]
Immunoassay	Fast, low cost, high sensitivity, excellent selectivity.	Weak anti-interference ability, poor stability, and precision.	[27]
Electrochemical method	Fast and convenient, low cost, excellent sensitivity.	Poor selectivity.	[28]

### 3.1 Derivatization method

The small molecular size of SEM means it lacks detectable UV and fluorescence chromophores. Its high water solubility and poor retention performance in reverse-phase chromatography make it susceptible to interference from coexisting small molecules during direct detection on mass spectrometers. The ionization efficiency on the ion source is low, and direct detection of secondary fragments is problematic, resulting in lower sensitivity. Therefore, derivatization analysis is an effective method to improve chromatographic retention performance and detection sensitivity. Currently, the detection of SEM primarily relies on high-performance liquid chromatography-tandem mass spectrometry (HPLC-MS/MS). In the newly released standard (GB-31656.13-2021), 2-nitrobenzaldehyde is used as the derivatizing agent, which requires shaking on a 37 °C shaker for 16 h. However, the prolonged hydrolysis and derivatization reaction time severely affect detection efficiency in practical work. To address this issue, many studies have reported on auxiliary hydrolysis and derivatization techniques to shorten reaction time. Relevant derivatizing agents and their derivatization methods are shown in Table 3.

**Table 3:** Assisted derivatization for SEM and their performance in analysis.

Samples	Derivatization agent	Molecular structure	Derivatization Method	Derivatization Condition	Detection method	LOD(μg/kg)	References
Shrimp	4-(acridone-10-yl)benzaldehyde		microwave-assisted derivatization	7.2 min,75 °C	HPLC-FLD	0.20-0.28	[29]
Fish	2-Nitrobenzaldehyde		microwave-assisted derivatization	2 h, 60 °C	UHPLC-DAD	0.25–0.33	[30]
Marine products	oxochromene-3-carbaldehyde (DAOC)		microwave-assisted derivatization	20 min,steam	HPLC-FLD	0.22–0.29	[31]
Fish, shrimp	2-Nitrobenzaldehyde		microwave-assisted derivatization	6 min,95 °C	LC-MS/MS	0.06	[32]
Fish, shrimp, chicken	4(N,Ndiphenylamino) benzaldehyde		ultrasonic-assisted derivatization	20 min,50 °C	UHPLC-FLD	0.21-0.28	[33]
Freshwater fish	2-Nitrobenzaldehyde		ultrasonic-assisted derivatization	1 h,40 °C	LC‐MS/MS	0.07-0.13	[34]
Shrimp	2-Nitrobenzaldehyde		microwave-assisted derivatization	30 min, 60 °C	UHPLC-MS/MS	0.20	[35]
Fish, shrimp	2-Nitrobenzaldehyde		conventional derivatization	Overnight, 37 °C	HPLC-MS	0.10-0.40	[36]
Fish	2-Nitrobenzaldehyde		rapid derivatization	5 min, 0 °C	UHPLC-MS/MS	0.40	[37]
Honey	5-nitro-2-furaldehyde		conventional derivatization	16 h, 37 °C	LC‐MS/MS	0.10-0.30	[38]

Wang et al. [33] have proposed a constant temperature ultrasound-assisted derivatization technique that can complete the derivatization of SEM in aquatic and livestock matrices within 20 min, with higher derivatization efficiency compared to heating derivatization. Veach et al. [32] used a combination of microwave derivatization, automatic solid-phase extraction, and LC-MS/MS technology to determine nitrofuran metabolites in aquatic products within 6 h. This procedure achieved accuracy between 89 and 107 % and relative standard deviation ≤ 8.3 %. Luo et al. [31] synthesized a new derivatizing agent, 4-(anthracen-10-yl)benzaldehyde. They used microwave-assisted derivatization to complete the derivatization of SEM in shrimp matrices within 7.2 min. It also resulted in a higher derivatization yield.

Presently, the majority of techniques employed to assess the use of nitrofurazone in animal food samples focus on the detection of its metabolite (SEM). Nevertheless, certain literature has reported the direct identification and quantification of nitrofurazone, as outlined in Table 4.

**Table 4:** The LODs of Analytical methods of NFZ.

Detection technology	LOD	Reference
HPLC	0.09 μg/kg	[39]
UV–visible spectroscopy	1.88 μM	[40]
Fluorescence	0.013 μM	[41]
Fluorescence	0.7 μM	[42]
Electrochemical	2.1 nM	[43]
Electrochemical	0.25 μM	[44]

### 3.2 Extraction purification

The SEM detection technique reported in the existing literature for aquatic products requires extraction and purification after derivatization to reduce interference from the sample matrix. Common extraction and purification techniques include liquid-liquid extraction and solid-phase extraction.

Derivatization increases the hydrophobicity of derivatized SEM. It is more soluble in organic solvents than in water-based solutions. This may be used to extract and separate water-soluble target molecules from aqueous impurities. Liquid-liquid extraction is a relatively simple and widely used method for detecting veterinary drug residues in aquatic products. In GB 31656.13-2021, the sample is hydrolyzed and derivatized, the pH is adjusted to neutral, and it is extracted with ethyl acetate, dried with nitrogen, and reconstituted. After purification with n-hexane for lipid and pigment removal, the sample is ready for detection. Similarly, Aldeek et al. [45] used ethyl acetate as the extractant and successfully applied liquid-liquid extraction to extract various nitrofurans metabolites, including SEM, from multiple seafood products, achieving recoveries between 90 and 100 % in spiked samples. Kim et al. [46] also used ethyl acetate as the extractant, extracted nitrofurans derivatives by liquid-liquid extraction, and purified them with a solid-phase extraction column. The detection was carried out under the minimum limit of detection (1 μg/kg) set by the European Union, and the recovery rate for SEM was 97.0 %–101.4 %.

Solid-phase extraction (SPE) is a method that integrates extraction, purification, and concentration into one, based on liquid-liquid extraction, with high separation efficiency, large sample volume, and low organic solvent usage. With the development of analytical techniques, solid-phase microextraction (SPME), matrix solid-phase dispersion extraction, and dispersed SPE have been extensively applied in the detection of SEM residues in aquatic products. Gong et al. [21] developed an inexpensive and easy-to-prepare wooden-tip SPME probe for determining SEM residues in large yellow croaker, achieving recoveries between 87.5 % and 112.7 %. This method shortened the sample purification time, reduced solvent consumption, and lowered testing costs. Tang et al. [47] prepared a new type of molecularly imprinted stirring rod for adsorbing and extracting SEM from fish samples. They combined it with high-performance liquid chromatography for detection, with recoveries between 96.2 % and 105.1 %. This method has the advantages of high extraction efficiency, simple operation, and low organic solvent consumption in sample pretreatment.

## 4 Instrumental analysis

Following the derivatization, extraction, purification, and concentration procedures, various instrumental analysis methods are commonly employed for SEM, including high-performance liquid chromatography (HPLC), liquid chromatography-tandem mass spectrometry (LC-MS/MS), immunoassay, and electrochemical analysis.

### 4.1 4.1.High-performance liquid chromatography

High-performance liquid chromatography (HPLC) is widely used in drug analysis due to its excellent ability to separate, identify, and quantify compounds. Various HPLC-based methods have been reported for detecting NFZ metabolites. Luo et al. [31] developed an HPLC-fluorescence detection method with microwave-assisted derivatization for determining nitrofuran metabolites in shrimp samples. A detection limit between 0.20 and 0.28 μg/kg was achieved. Yu et al. [48] established a simple and sensitive HPLC-fluorescence detection method with a limit of detection between 0.20 and 0.30 μg/kg for detecting metabolites in shrimp samples. Du et al. [49] simultaneously detected four nitrofuran metabolites in shrimp using HPLC-fluorescence detection, with a detection limit of 0.20–0.26 μg/kg.

### 4.2 2.Liquid chromatography-tandem mass spectrometry

The liquid chromatography-tandem mass spectrometry (LC-MS/MS) analysis method has excellent separation capability and superior qualitative and quantitative analysis performance and is widely used in the analysis of nitrofuran metabolites. The latest standard, GB-31656.13-2021 clearly specifies the use of LC-MS/MS to detect nitrofuran metabolite residues in processed aquatic products [50]. Guichard et al. [51] developed an LC-MS/MS method for determining nitrofuran metabolites in meat and aquatic products, with the detection limit ranging from 0.03 to 0.23 μg/kg. Wu et al. [37] used UPLC-MS/MS to detect nitrofuran metabolites in fish, with a sensitivity of up to 0.40 μg/kg. Melekhin et al. [38] used 5-nitro-2-furaldehyde as a derivatization reagent combined with LC-MS/MS to detect nitrofuran metabolites in honey, with the detection limit ranging between 0.10 and 0.30 μg/kg. These methods are of certain reference value for relevant work.

### 4.3 3.Immunoassay

The immunoassay has several advantages such as fast detection, low cost, high sensitivity, and strong selectivity, making it widely used in detecting various drug residues in food. Additionally, it has been widely applied in the research of SEM detection methods. Dong et al. [27] utilized Eu(III) nanoparticles mediated by sheep anti-mouse immunoglobulin to perform specific and multiple detections of nitrofuran metabolites, which could produce detection results that can be observed with naked eye within 10 min at the detection limit of 0.10 μg/kg. Yu et al. [52] established a biomimetic enzyme-linked immunosorbent assay based on molecular imprinting technology to detect SEM in shrimp, with a 1.00 μg/kg detection limit. Wang et al. [53] achieved rapid and multiple immunochromatographic detection of nitrofuran metabolites in fish samples, within 15 min. The detection limit for SEM is 0.75 ng/mL. Wu et al. [54] prepared a colloidal gold-labeled antibody immunoprobe, which can quickly determine SEM in fish and shrimp, with the limit of detection of 0.12 and 0.15 μg/kg, respectively.

### 4.4 4.Electrochemical detection

The electrochemical detection technology is a rapid and straightforward method that relies on interface reactions and signal recognition sensing. It offers high accuracy, sensitivity, and reproducibility and has been employed in the rapid analysis and detection of SEM. Yu et al. [55] used a composite material consisting of a molecularly imprinted polymer, carboxylated single-walled carbon nanotube, and chitosan as an electrochemical sensing interface to detect SEM with the detection limit at 0.025 ng/mL. He et al. [56] developed an electrochemical sensor by modifying a thin film gold electrode with a nanogold/graphene composite for the detection of NFZ and SEM. The detection limit for NFZ was 0.13 μmol/L, while for SEM, it was 0.0047 μmol/L.

## 5 New metabolic marker of NFZ

Extensive research has demonstrated that SEM can be endogenously produced in aquatic crustaceans [57]. Many crustacean products contain background residues of SEM. Although residual SEM in crustacean aquatic products may be related to endogenous substances, existing detection techniques cannot distinguish between endogenous and exogenous SEM, and the mechanism of endogenous SEM formation remains unclear. As explicitly stated in the latest GB31656.13–2021, SEM is not suitable as a marker for monitoring the illegal use of NFZ drugs in crustacean aquatic products. Therefore, the determination of SEM residues in crustacean products such as shrimp and crab is excluded.

**Fig. 4:**
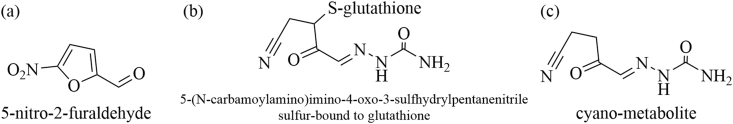
Molecular structure of nitrofurazone potential metabolites.

In recent years, researchers have proposed other potential NFZ metabolites, besides SEM, as a basis for detecting the illegal use of NFZ [[58], [59], [60]]. These metabolites include 5-nitro-2-furaldehyde(5-NF), 5-nitro-2-furaldehyde-2,4-dinitrophenylhydrazone, and the complex formed by glutathione and NFZ metabolites, as shown in Fig. 4(a-c). Among them, 5-NF is the most frequently mentioned potential metabolite, but its detection is challenging due to its photosensitivity and air sensitivity [61]. However, several researchers have developed highly efficient liquid chromatography detection methods based on the reaction of 5-NF with 2,4-dinitrophenylhydrazine to detect it in shrimp and crab. For instance, Zhang et al. [62] successfully detected and quantified 5-NF in aquatic products using ultra-high-performance liquid chromatography-tandem mass spectrometry with the limit of detection and quantification limit below 1 μg/kg. Wang et al. [63] established a method for determining 5-NF in shrimp by ultra-high-performance liquid chromatography-tandem mass spectrometry with a detection limit at 0.05 μg/kg.

Furthermore, researchers have found that the complex formed by glutathione and NFZ metabolites can be used as a new marker for monitoring the illegal use of NFZ in crustacean aquatic products [60]**.** Additionally, after feeding catfish with NFZ, Wang et al. observed that the cyanide metabolite (Fig. 4c) had a half-life elimination of 81 h in muscle and a residual period of over 14 d, demonstrating its feasibility as a new marker for determining the illegal use of NFZ in aquatic products [64].

Overall, the background residue of SEM in crustacean products such as shrimp and crab under GB31656.13–2021 has led researchers to explore other potential NFZ metabolites as markers for monitoring the illegal use of NFZ in crustacean aquatic products.

## 6 Summary and prospect

SEM has multiple sources, and metabolites of the veterinary drug NFZ are not its sole origin. Up-to-date, SEM has been detected in different types of food (including animal and non-animal sources) and even in the environment, raising concerns about food safety. Although a range of detection techniques for SEM has been developed domestically and internationally, these methods are still unable to identify the source of SEM in samples. Moreover, the high fat and protein content in aquatic products require complicated pretreatment procedures for on-machine detection, resulting in low efficiency. Currently, high-performance liquid chromatography-tandem mass spectrometry is the mainstream method for SEM detection. In contrast, immunoassay methods are often used as a rapid screening method in laboratories or monitoring sites without liquid chromatography-mass spectrometry conditions. Other detection methods have certain limitations in accuracy and are less frequently used in practical applications. In future research on technology for regulating the illegal use of NFZ, there are still some issues that need to be addressed.

i The false positives caused by endogenous SEM are becoming increasingly problematic. There is a lack of accurate identification of its source, which seriously hinders regulatory efforts to combat the illegal use of NFZ. Finding a solution to this problem is critical.
ii The pretreatment techniques for SEM detection are still relatively complex, time-consuming, and labor-intensive. Further research is needed to develop more environmental-friendly, cost-effective, and dependable pretreatment materials and techniques to improve detection efficiency.
iii Endogenous SEM is found in varying levels in different tissues of aquatic crustacean products, and its formation mechanism is not yet clear. In-depth research is needed to understand the formation of endogenous SEM. Differentiating between endogenous and exogenous SEM is important from a source perspective.
iv Although detection methods using new metabolite markers, such as 5-NF for NFZ, have been proposed in existing research, these methods are not yet mature and require further optimization. Expanding their use in actual samples and developing them into stable and reliable detection methods can provide crucial evidence to address the problem of false positives caused by endogenous SEM in the illegal use of NFZ.

## Data availability statement

The authors declare that no data associated with our study has been deposited into a publicly available repository. Data are included and referenced in the article.

## CRediT authorship contribution statement

**Guangxin Yang:** Conceptualization, Data curation, Investigation, Writing – original draft, Writing – review & editing, Validation. **Shuhai Ding:** Data curation, Investigation, Validation, Writing – original draft, Methodology. **Junyu Zhang:** Conceptualization, Data curation, Methodology. **Lin Gu:** Methodology, Writing – review & editing. **Wenlei Zhai:** Investigation, Writing – review & editing. **Cong Kong:** Conceptualization, Funding acquisition, Investigation, Project administration, Resources, Validation, Writing – review & editing.

## Declaration of competing interest

The authors declare that they have no known competing financial interests or personal relationships that could have appeared to influence the work reported in this paper.
